# Supplementary material for: Patterns of Intron Gain and Loss in Fungi
Source: PLoS Biol. 2004 Nov 30;2(12):e422. doi: 10.1371/journal.pbio.0020422 (PMC532390; doi:10.1371/journal.pbio.0020422)
Supplement: Table S1 — Also available at http://genes.mit.edu/NielsenEtAl/. (4.3 MB ZIP). [file pbio.0020422.st001.zip › NielsenEtAl/html/196.html]

AN1915.1.NCU04817.1.MG04969.1.FG08760.1


```
 CLUSTAL W (1.82) Multiple Sequence Alignments - Introns Inserted


Sequence 1: AN1915.1	441 aa
Sequence 2: NCU04817.1	492 aa
Sequence 3: MG04969.1	510 aa
Sequence 4: FG08760.1	479 aa
Alignment Length: 518 aa
Number Identitical Residues: 214 aa
Alignment Score (without introns) 10367


MG04969.1 	-MSLYRIGIPRTLGRLSSPAFVCRQCLHTQPLKASQVARQVIRSPALSRNQTSTSTSTGN
NCU04817.1	-MAFFQLGLRRAAVQLSRPSVFC----ARAAFNASQQQKLVQLATPASTILKLVSQARHT
FG08760.1 	MASSFAPGFRRLL--------------FQASIKASGPSKFFVCNQCLRTAPRSMPSRVLN
AN1915.1  	---------------------------MDATF--------------------LRSKARKA
          	                              .:                      .     

MG04969.1 	NARTKWLVGGSRTFFRSGTKSSSGSTATAVEAATAEASSEAGAGARSSSFPETNSKAVGY
NCU04817.1	QSRN----------YATQKVQESPLSKLATDIAKPAPSSDKSE--KKSSFPETSSKSVAY
FG08760.1 	AVRSRS--------YADAAVSKPIGSIAEQASVHASPSVSQAA---KKAWPESNPKGVGL
AN1915.1  	FAR-----------YASDSA--------AVNGAKPEAS--------KSAFPDVSSKSVAY
          	  *           :                 . . .*        ..::*: ..*.*. 

MG04969.1 	WLIGSAVSVFGIVIFGGLTRLTESG2LSITEWRPVTGSLPPRNAEDWESEFSKYRASPEF
NCU04817.1	WLLASAASVFGIVIFGGLTRLTESG2LSITEWRPVTGSLWPGSQEEWESEFEKYRASPEF
FG08760.1 	WLIGSAVSVFGIVVFGGLTRLTESG2LSITEWRPVTGSLPPMSKEDWESEFEKYRASPEF
AN1915.1  	WLLASAASVFGIVVFGGLTRLTESG2LSITEWRPVTGSLPPMNAEDWESEFSKYRASPEF
          	**:.**.******:*********** ************* * . *:*****.********

MG04969.1 	ALLNPHMTLDEFKKIYYMEWTHRLWGRFIGLTFVLPTVYFIARKQVTRKMAFNLVGISAL
NCU04817.1	KMLNSTMNLQEFKQIYFMEWTHRLWGRFIGMSFVLPTIYFIARRRVTPRMACTLVGISAL
FG08760.1 	KLLNPHMDLEEFKKIYFMEWFHRVWGRFIGLTFVIPTAYLVARRKVTPKMALNLAGISAL
AN1915.1  	QLLNPTMTLSEFKSIYYMEWIHRLWGRFVGLSFVLPAVYFVARKKVSKPMVLRLGGIAGL
          	 :**. * *.***.**:*** **:****:*::**:*: *::**::*:  *.  * **:.*

MG04969.1 	IGFQGFIGWWMVKSGLKDDLFAPGSHPRVSQYRLTTHLGTAFLCYSAMLLSGLSVLRTRR
NCU04817.1	IGFQGFIGWWMVKSGLKDDLFAPGSHPRVSQYRLAAHLATAFVCYSWMLLAGLSILRTHK
FG08760.1 	IGFQGIIGWWMVKSGLKDDLFAPGSHPRVSQYRLTTHLATAFVCYSWMLVSGLTVLRTRR
AN1915.1  	IGFQGFIGWWMVKSGLKDDLFAPGSHPRVSQYRLTAHLGAAFVCYTAMLWNGLAILRSHR
          	*****:****************************::**.:**:**: **  **::**:::

MG04969.1 	LVADPTAATKLFSQLKNAAITPFRRYVLAMTALVFTTAMSGALVAGLDAGLIYNEFPKMG
NCU04817.1	AINFPEQAVKNFTALQSPALRVLRGSVAALTVLIFTTVLSGALVAGLDAGLIYNEFPMMG
FG08760.1 	WLMNPEAAMKQIGEMSQPGLRTMRRVVFALAALTFTTAMSGGLVAGLDAGLIYNEFPKMG
AN1915.1  	LLSDPEAGLKLLDSLKDPKLKLFRRSVAGLALLVFVTAMSGALVAGLDAGLIYNEFPWMG
          	 :  *  . * :  :... :  :*  * .:: * *.*.:**.*************** **

MG04969.1 	VGLTPPMTELFDKFYSRKEDGSDLWWRNMLENPSLVQLDHRALAMTTFCAVVAMFVWSR-
NCU04817.1	NGLAPPKSELLDKFYSRHEDGSDLVWRNMLENPSLVQLDHRILAMTTFTAVCALVAYSR-
FG08760.1 	LGIFPPKQELFDKFYSRKEDHSDLWWRNMLENPSTVQMNHRILAVTTFTSILSLFIYAR-
AN1915.1  	NGLAPPMSELFDDRYSRHEDRSDRWWRNMLENPSLVQLDHRILAMTTFTSIMALWAYTRR
          	 *: **  **:*. ***:** **  ********* **::** **:*** :: ::  ::* 

MG04969.1 	--RLGAVLPTSARKGSMGVLHLALIQVALGITTLIYMVPTHLAATHQAGALALLTGSLVL
NCU04817.1	SGRVKAALPPAARKGITGLLHLVSLQVALGISTLIYMVPIPLAAAHQAGALALLTGVLVA
FG08760.1 	TRRVKAILPTNIKKGANGLFHMVSLQVALGISTLIYMVPISLAAAHQAGALGVLTMAIVL
AN1915.1  	SPNMKRLLPPSAKKGVHGVVAFACMQVGLGLTTLLYLVPTPLASAHQAGSLFLLTWVLVL
          	: .:   **.  :**  *:. :. :**.**::**:*:**  **::****:* :**  :* 

MG04969.1 	SHRLHVS----KSTVSLIQRQLLKSAGSGGAARLPTKRI
NCU04817.1	GQRLRIP----KATLAMAQRRLAQLAQ---QAKTQGPK-
FG08760.1 	AHRLHVP----KPTLRLLEKRLKAAAA---AAK------
AN1915.1  	GSRVWHPSRTAKLLQLAAKARGNSLPN---VTRQAAPKL
          	. *:  .: ::*      : :    .     ::
```
